# Sex-specific epigenetic signatures of circulating urate and its increase after BCG vaccination

**DOI:** 10.21203/rs.3.rs-4498597/v1

**Published:** 2024-07-22

**Authors:** Zhaoli Liu, Tania O. Crișan, Cancan Qi, Manoj Kumar Gupta, Xuan Liu, Simone J.C.F.M. Moorlag, Valerie A.C.M. Koeken, L. Charlotte J. de Bree, Vera P. Mourits, Xu Gao, Andrea Baccarelli, Joel Schwartz, Frank Pessler, Carlos A. Guzmán, Yang Li, Mihai G. Netea, Leo A.B. Joosten, Cheng-Jian Xu

**Affiliations:** 1 Centre for Individualised Infection Medicine (CiiM), a joint venture between the Helmholtz-Centre for Infection Research (HZI) and Hannover Medical School (MHH). Hannover, Germany.; 2 TWINCORE, a joint venture between the Helmholtz-Centre for Infection Research (HZI) and Hannover Medical School (MHH). Hannover, Germany.; 3 Department of Internal Medicine and Radboud Center for Infectious Diseases (RCI), Radboud University Medical Center. Nijmegen, the Netherlands.; 4 Department of Medical Genetics, „Iuliu Hațieganu” University of Medicine and Pharmacy. Cluj-Napoca, Romania.; 5 Department for Immunology and Metabolism, Life and Medical Sciences Institute (LIMES). University of Bonn. Bonn, Germany.; 6 Department of Environmental Health, Mailman School of Public Health, Columbia University, New York, NY, USA.; 7 Department of Occupational and Environmental Health Sciences, School of Public Health, Peking University, Beijing, China.; 8 Harvard T.H. Chan School of Public Health, Boston, MA, USA.; 9 Department of Environmental Health, Harvard T.H. Chan School of Public Health, Boston, MA, USA.; 10 Research Group Biomarkers for Infectious Diseases, TWINCORE, a joint venture between the Helmholtz-Centre for Infection Research (HZI) and Hannover Medical School (MHH), Hannover, Germany.; 11 Department Vaccinology and Applied Microbiology, Helmholtz-Centre for Infection Research (HZI), Braunschweig, Germany.; 12 Research Centre Innovations in Care, Rotterdam University of Applied Sciences, Rotterdam, the Netherlands.; 13 Cluster of Excellence RESIST (EXC 2155), Hanover Medical School, Hannover, Germany.; 14 Lower Saxony center for artificial intelligence and causal methods in medicine (CAIMed). Hannover, Germany.

**Keywords:** serum urate, DNA methylation, BCG vaccination, urate change, sex-specificity

## Abstract

**Background:**

Urate concentration and the physiological regulation of urate homeostasis exhibit clear sex differences. DNA methylation has been shown to explain a substantial proportion of serum urate variance, mediate the genetic effect on urate concentration, and co-regulate with cardiometabolic traits. However, whether urate concentration is associated with DNA methylation in a sex-dependent manner is unknown. Additionally, it is worth investigating if urate changes after perturbations, such as vaccination, are associated with DNA methylation in a sex-specific manner.

**Methods:**

We investigated the association between DNA methylation and serum urate concentrations in a Dutch cohort of 325 healthy individuals. Urate concentration and DNA methylation were measured before and after *Bacillus Calmette-Guérin* (BCG) vaccination, used as a perturbation associated with increased gout flares. The association analysis included united, interaction, and sex-stratified analysis. Validation of the identified CpG sites was conducted using three independent cohorts.

**Results:**

215 CpG sites were associated with serum urate in males, while 5 CpG sites were associated with serum urate in females, indicating sex-specific associations. Circulating urate concentrations significantly increased after BCG vaccination, and baseline DNA methylation was associated with differences in urate concentration before and after vaccination in a sex-specific manner. The CpG sites associated with urate concentration in males were enriched in neuro-protection pathways, whereas in females, the urate change-associated CpG sites were related to lipid and glucose metabolism.

**Conclusion:**

Our study enhances the understanding of how epigenetic factors contribute to regulating serum urate levels in a sex-specific manner. These insights have significant implications for the diagnosis, prevention, and treatment of various urate-related diseases and highlight the importance of personalized and sex-specific approaches in medicine.

## Graphical Abstract

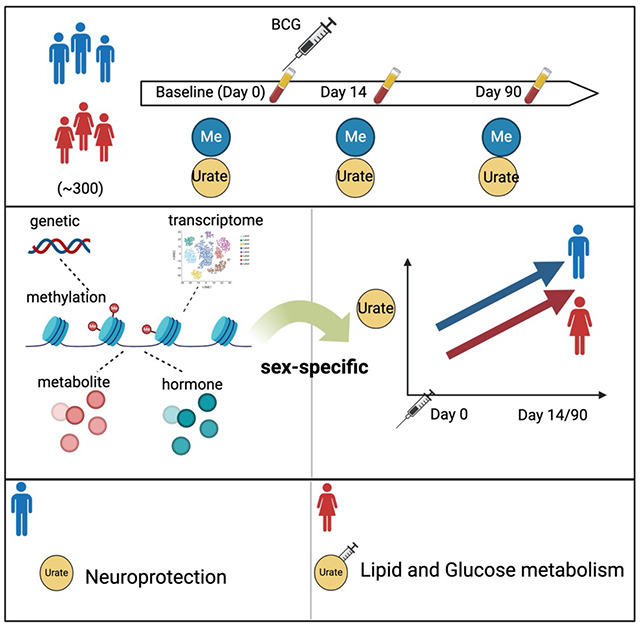

## Introduction

Urate is the end product of purine metabolism in humans (1,2) and has been shown to have beneficial effects, including acting as an antioxidant (3), enhancing the immune response (4), and reducing the risk of neurodegenerative disease (5). However, an inappropriately elevated concentration of urate, known as hyperuricemia, can lead to the development of gout and other health conditions (6–8). Therefore, elucidating the regulatory mechanisms governing circulating urate concentration is crucial.

Previous research has identified both host and environmental factors that can influence circulating urate concentration (8,9). While a genome-wide association study (GWAS) has identified 183 loci associated with urate concentration, these loci only explained about 7.7% of the variance in serum urate concentration (10), suggesting that other non-genetic factors may play an important role. DNA methylation is one of the well-studied epigenetic markers (11), and modifications to DNA methylation record an individual’s response to environmental exposures (12). A recent large-scale trans-ancestry epigenome-wide association study (EWAS) identified 99 urate-associated Cytosine-phosphate-Guanine (CpG) sites, which collectively account for 11.6% of serum urate variance (12). This underscores the crucial role of epigenetic regulation in urate concentration. While sex differences in circulating urate concentrations and their association with genetic, cardiometabolic traits, and sex hormones are well studied, the extent to which the epigenome is differentially associated with urate concentrations in males and females is not well understood.

Vaccines have been widely used to prevent infections, yet recent reports suggest that vaccination might increase the risk of gout flares (13,14). Gout is the main disease which is primarily associated with hyperuricemia (15,16). However, whether vaccination impacts circulating urate concentrations or the exact mechanism by which they could drive gout flares remains unknown. In this report, we focus on the *Bacillus Calmette-Guérin* (BCG) vaccine, commonly used as an anti-tuberculosis vaccine, to investigate the epigenetic mechanisms of interactions between vaccination and urate metabolism. Prior studies have indicated an association between anti-tuberculosis drugs and hyperuricemia (17,18), prompting the exploration of whether the BCG vaccine, as an anti-tuberculosis vaccine, is similarly associated with increased urate levels. BCG vaccination may potentially promote a proinflammatory environment, thereby contributing to the development of inflammation-mediated diseases. Moreover, it induces trained immunity, which could modulate the immune response in gout and inflammasome activation triggered by urate and urate crystals (15,19).

To investigate the interplay among epigenetic signatures, circulating urate concentrations, and vaccination, we recruited 325 Dutch individuals from the Human Functional Genomics Project (300BCG cohort). Genome-wide DNA methylation profiles and urate concentrations were measured for all participants before BCG vaccination, as well as two weeks and three months after vaccination. We conducted the first sex-specific epigenome-wide association study (EWAS) of urate concentration and vaccination-induced urate changes, respectively, including CpG sites from both autosomes and the X chromosome. Utilizing baseline data before vaccination, we identified a sex-specific association between serum urate and DNA methylation. Specifically, in males, urate-associated CpG sites were predominantly enriched in genes linked to pathways such as neuroprotection. After BCG vaccination, we observed a significant increase in serum urate concentration at 2 weeks, which remained elevated for up to three months. Moreover, we observed a sex-specific epigenetic association with BCG-induced urate changes. In females, urate change-associated CpG sites were enriched in genes related to lipid and glucose metabolism. These identified CpG sites were further validated in three additional cohorts. Our study provides valuable insights into how epigenetic associated with urate concentration before and after vaccination, which may enable us to better understand sex-specific epigenome co-regulation of urate and other urate-related diseases.

## Results

### Cohort information and study design

**Figure 1.**
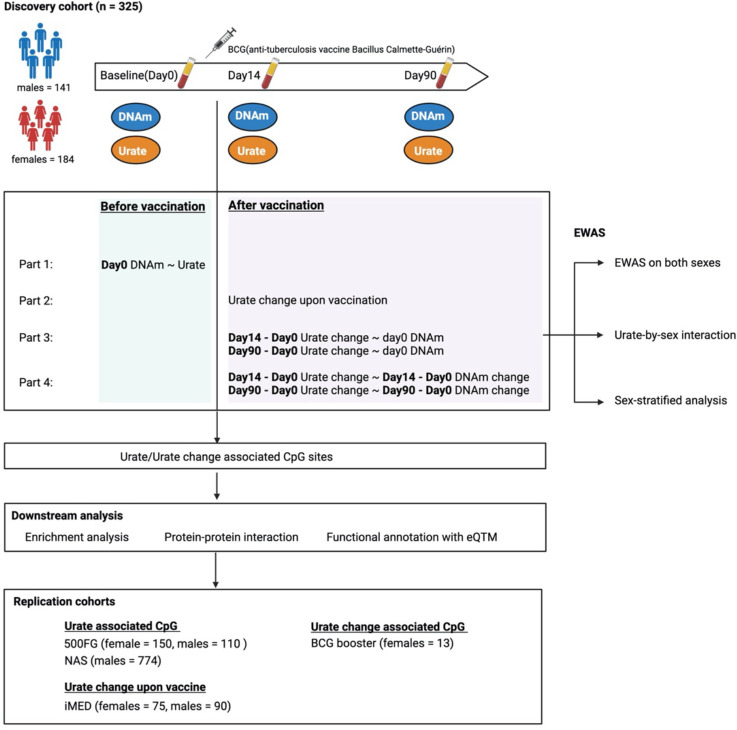
The workflow of this EWAS on urate concentrations. In this study, we included one discovery cohort called 300BCG, DNA methylation (DNAm) and urate data were generated from this cohort. We did the following analyses in our study. First, we explored whether the baseline serum urate was associated with baseline DNA methylation in a sex-specific way by interaction and sex-stratified analysis. Second, we investigated the urate change and performed the EWAS of urate change after BCG. We did the EWAS on both sexes, urate-by-sex interaction EWAS, and sex-stratified EWAS. After obtaining the urate or urate change-associated CpG sites, we annotated these sites with their proximal genes, followed by enrichment analysis and protein-protein interaction analysis. We also linked these CpG sites with gene expression using published eQTM results. Independent cohorts were used to validate the identified CpG sites.

Our discovery cohort (300BCG (20–23)) involved 325 Dutch individuals (56% female). Measurements were taken at three-time points: baseline, 14 days after, and 90 days after BCG vaccination. We assessed whole blood genome-wide DNA methylation (DNAm) of 850,000 CpG sites and serum urate concentrations (Figure 1, **Figure S1, Table S1**). To replicate significant associations between urate concentrations and DNA methylation, we included three additional cohorts (500FG (24,25), n=260; NAS (26), n=774; BCG booster (27), n=13) as replication cohorts, in which both methylome, urate levels and BCG induced urate changes were measured. Details on these cohorts are provided in the Methods section. The analysis workflow is presented in Figure 1.

### Discovery and replication of sex-specific urate-associated CpGs at baseline

**Figure 2.**
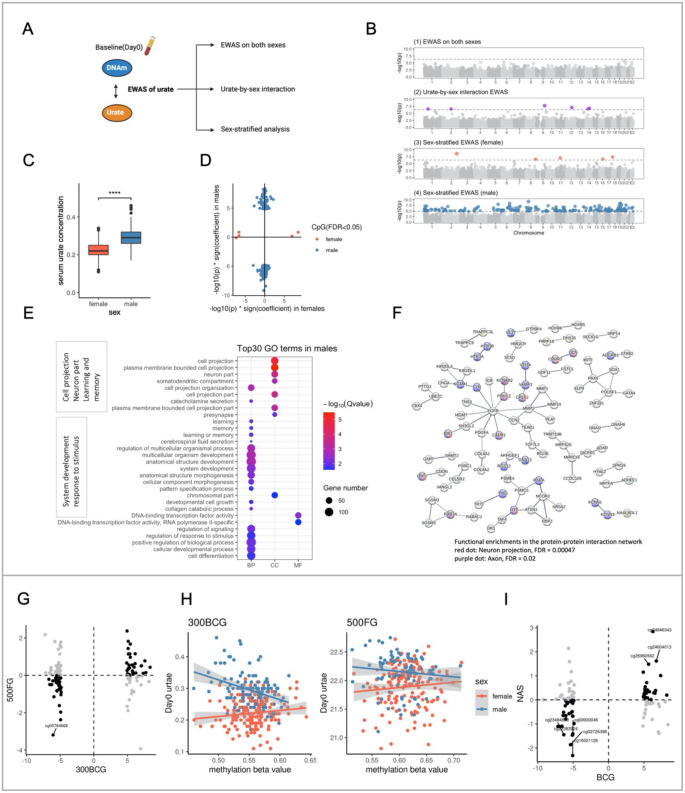
Serum urate concentrations were associated with DNA methylation in a sex-specific manner. (A) Schematic representation of the analysis flow. (B) The Manhattan plot shows the results from EWAS of urate in all participants (1), urate-by-sex interaction analyses (2), females (3), and males (4) respectively. The CpG sites were ordered by their chromosomal position on the x-axis with their −log10(P value) of the association on the y-axis. The dotted horizontal lines represented the level of significance corrected for multiple testing. colorful dot: significant CpG sites with FDR < 0.05, grey dot: non-significant CpG sites. (C) Bar plot showing the difference of the serum urate level at baseline between males and females. (D) The scatter plot showing the distribution of −log10(P value) * sign(effect estimate) of the significant sites (FDR < 0.05) from urate-EWAS in males and females. The color represented the sites from different sex groups. (E) Dot plot describing the GO enrichment categories of genes annotated to the significant CpG sites identified in males. The color and size of the dot indicate the significance and the number of annotated genes in the given GO term respectively (BP: biological process, MF: molecular function, CC: cellular component). The top 30 enriched categories were shown. (F) Protein-protein interaction networks functional enrichment analysis of the urate-associated CpG sites from males. (G) Scatter plot showing the distribution of −log10(P value) * sign (effect size) of the male urate-associated CpG sites in BCG and 500FG cohorts. Black dots represented CpG sites with the same effect direction, while grey dots represented CpG sites with different effect directions. (H) One example of the replicated CpG site. (I) Scatter plot showing the distribution of −log10(P value) * sign (effect size) of the male urate-associated CpG sites in BCG and NAS cohorts. Black dots represented CpG sites with the same effect direction, while grey dots represented CpG sites with different effect directions.

Univariate associations between principal components (PCs 1–20) derived from the DNA methylation data and covariates, including estimated cell proportion of six cell types by Houseman’s method (28) are shown in **Figure S2**. The top 20 PCs collectively accounted for 30.34%, 30.17%, and 33.70% of the variance in DNAm at baseline, 14 days, and 90 days post BCG vaccination, respectively. Cell proportions, batch (sample plate), and age were significantly associated with the top 5 PCs, whereas smoking status, BMI, height, and weight did not show any significant correlation (**Figure S2**). Next, we assessed the association of urate concentration with DNA methylation in all participants in the 300BCG cohort at baseline (day 0) (Figure 2A). Although no genome-wide significant CpG site was identified (False Discovery Rate (FDR) adjusted P value < 0.05) (Figure 2B (1)), 81 out of 96 urate-associated CpG sites reported previously (12) exhibited a consistent direction of the estimated effect (**Figure S3A**).

Given the significant difference in baseline urate concentrations between males and females observed (P value < 2.2e-16, Wilcoxon rank sum test, Figure 2C), we examined the influence of sex on the DNA methylation-urate association. We employed two approaches: urate-by-sex interaction analysis and sex-stratified analyses. The interaction analysis identified six CpG sites exhibiting significant interaction effects (Figure 2B (2), **Figure S3B**). The CpG site with the strongest interaction effect was cg03227576 (P value = 1.95e-08), mapping to the *PAPPA* gene, which encodes a protein involved in inflammation (29), systemic glucose homeostasis (30), and female fertility (31). Intriguingly, sex-stratified analyses revealed 215 genome-wide significant CpG sites in males compared to only 5 CpG sites in females (Figure 2B (3–4), **Table S2–3**). This finding may suggest a potential sex-specific epigenetic association of urate concentrations. The comparable inflation factors for both models (1.04 in females vs 1.11 in males) indicate that this difference is unlikely to be due to model inflation. Furthermore, Figure 2D highlights the distinct effects between males and females. Gene enrichment analysis of the 215 urate-associated CpG sites annotated genes in males revealed enrichment in pathways related to cell projection, neuron part, learning and memory, system development, and response to stimulus terms (Figure 2E). Consistent with this, the protein-protein interaction network analysis based on genes annotated to significant CpG sites in males showed functional enrichments in the neuron projection and axon (cellular component in Gene Ontology) (Figure 2F). When we checked the association between identified CpG sites and gene expression using the Biobank-based integrative omics study (BIOS) database (32) (**Table S4**), we found that cg05231308 significantly correlated with *UCN* gene expression, which is implicated in both neurodegenerative conditions and skeletal system disorders. Additionally, cg09930046 correlated with the expression of *AZU1*, a crucial player in innate immunity, functioning as both a neutrophil granule-derived antibacterial factor and a monocyte- and fibroblast-specific chemotactic glycoprotein (33). Conversely, protein-protein interaction analysis in females showed enrichment in cell cycle-associated pathways.

We sought to validate the urate-associated CpG sites identified in males from the 300BCG cohort using two independent cohorts: the 500FG (n=260) and the NAS cohort (n=774). In the 500FG, 72 out of 130 CpG sites showed the same direction of association with urate concentrations, with eight sites reaching nominal significance (P value < 0.05). One CpG cg00764668 achieved an FDR significance (FDR = 0.042) and is located on chr19, mapping to the *KCNN1* gene (Figure 2G). *KCNN1* is associated with intellectual developmental disorder, potassium channels, and transmission across chemical synapses (34). Notably, the methylation level at cg00764668 exhibited a negative correlation with circulating urate concentration in males but not in females (Figure 2H). In the NAS, 57 out of 107 CpG sites showed the same direction, with eight sites reaching nominal significance and no CpG site reaching FDR significance (Figure 2I). Next, when we validated the findings of urate-DNA methylation in males by meta-analysis with 500FG and NAS, 12 out of 63 CpG sites showed the same direction across all these three cohorts, including the cg00764668 mentioned above. Two CpG sites, cg22383924 (mapped to gene *TP73*) and cg24846343 (mapped to gene *DDTL*), reached nominal significance (**Table S5**). cg22383924 annotated to gene *TP73*, which plays a role in cellular responses to stress and development (35). Replication of CpG sites identified in females from the 300BCG cohort was performed using the 500FG cohort. Two out of four CpG sites displayed the same direction of association, with one site (cg00864618) reaching nominal significance. cg00864618 mapped to gene *CHRAC1*, which plays a role in DNA transcription, replication, and packaging with other histone-fold proteins (36).

### Discovery and replication of sex-specific CpGs associated with BCG-induced urate change

**Figure 3.**
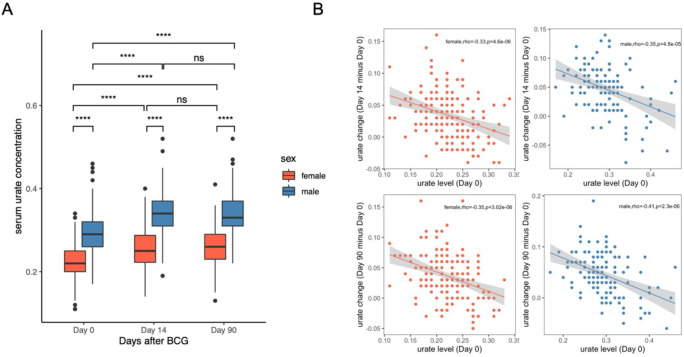
Serum urate significantly increased after BCG vaccination and ΔUrate was negatively correlated with baseline urate. (A) Boxplot showing the serum urate concentration at different time points, color indicating different sex groups. The paired samples Wilcoxon test was used to compare the urate concentration among different time points. Wilcoxon rank sum test was used to compare the difference between males and females. ns: not significant, *: P < 0.05, **: P < 0.01, ***: P < 0.005, ****: P < 0.001. (B) Spearman’s correlation between ΔUrate and Urate_baseline_. The color indicated different sex groups.

As shown in Figure 3A, BCG vaccination induced a significant increase in urate concentrations (median value in females: Urate_baseline_=0.22, Urate_day14_=0.25, P value < 2.2e-16; median value in males: Urate_baseline_=0.29, Urate_day14_=0.34, P value < 2.2e-16. Paired samples Wilcoxon test). Notably, these elevated urate concentrations persisted for up to three months after vaccination (median value in females: Urate_day90_=0.26; median value in males: Urate_day90_=0.33) (Figure 3A). Interestingly, the magnitude of the urate rise differed by sex, with males exhibiting a higher increase in both short-term (P value = 0.00058, One-sided Wilcoxon rank sum test) and long-term (P value = 0.01, One-sided Wilcoxon rank sum test) than female (**Figure S4A**). Both short-term and long-term urate change showed a significantly negative correlation with Urate_baseline_ in both sexes (Figure 3B).

**Figure 4.**
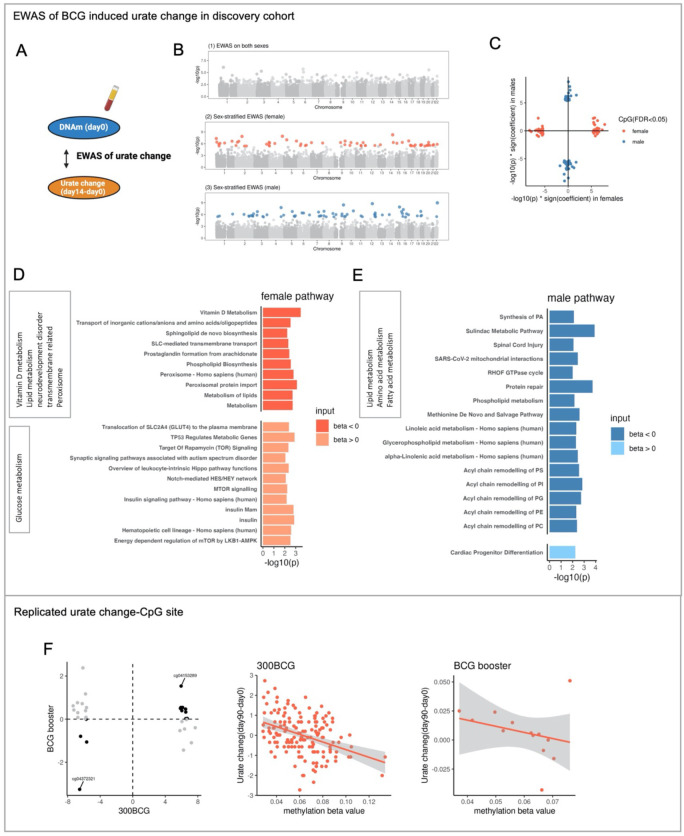
ΔUrate associated with DNAm_baseline_ in a sex-specific manner and urate change-associated CpG sites were related to human metabolism. (A) Schematic representation of the analyses. (B) The Manhattan plot showing the results from EWAS of Δurate_short_ in all participants (1), females (2), and males (3) respectively. The CpG sites were ordered by their chromosomal position on the x-axis with their −log10 (P value) of the association on the y-axis. Colorful dot: significant CpG sites, grey dot: non-significant CpG sites. (C) The scatter plot showing the distribution of −log10 (P value) * sign(effect estimate) of the significant sites from EWAS of Δurate_short_ in males and females. Color represented the significant sites from different sex groups. (D-E) Bar plot describing the KEGG enrichment categories of genes annotated to the significant CpG sites identified from the EWAS of ΔUrate_short_ in females (D) or males (E). “BETA < 0” represented the significant sites with a negative effect estimate. “BETA > 0” represented the significant sites with a positive effect estimate. (F) One example of the replicated urate change-associated CpG site. The y-axis represented the urate change from day90 minus day0 and after rank normalization. The x-axis represented the beta value of cg04372321.

Next, we assessed the association between baseline DNA methylation and BCG-induced urate change in the 300BCG cohort (Figure 4A). We first conducted an EWAS between baseline DNAm and urate change at 14 days post-vaccination (short-term change) for all participants. This analysis did not identify any epigenome-wide association CpG sites (Figure 4B (1)). As urate change after BCG vaccination differed between males and females, we stratified the participants by sex and identified sex-specific CpG sites associated with urate change: 63 in females and 67 in males (Figure 4B (2–3)). Notably, the effect size patterns of these CpG sites were distinct between males and females (Figure 4C), suggesting sex-based differences in epigenetic associations. The CpG site with the strongest association in females was cg21182196 (P value = 5.46e-09), mapping to the *CLMN* gene. This gene might be involved in neuron projection development and its expression could be regulated by vitamin D3 (37). In males, the most significant CpG site was cg03088047 (P value = 1.06e-09), mapping to *TBC1D22A*, a gene involved in regulating lipid homeostasis (38).

In females, urate change-associated CpG sites were linked to genes involved in vitamin D metabolism, lipid and glucose metabolism, and SLC-mediated transmembrane transport processes (Figure 4D, **Table S6**). Among the 34 CpG sites with a negative association with urate increase, 13 were mapped to the genes related to metabolism, such as genes in the lipid metabolism pathways (*PCYT1A*; *ALDH3B2*; *DHCR7*; *SPNS2*; *GC*; *PGS1* and *SETD4*). Notably, 17 CpG sites were annotated to genes in the SLC-mediated transmembrane transport process and peroxisome pathway. On the other hand, the 29 CpG sites with a positive association with urate increase were annotated to the genes involved in the insulin signaling pathway (*PRKAG2*; *RHEB*; *YWHAQ* and *SOCS1*). Interestingly, pathways related to the translocation of SLC2A4 (GLUT4) to the plasma membrane were enriched, suggesting these sites were linked to glucose metabolism. Additionally, the enrichment of synaptic signaling pathways associated with autism spectrum disorder points to potential neuromodulatory roles (Figure 4D, **Table S6**). In males, the genes annotated to those CpG sites with a positive correlation with urate increase were enriched in the cardiac progenitor differentiation pathway. Conversely, CpG sites with a negative correlation with urate increase were linked to various metabolic pathways, including amino acid metabolism (Methionine De Novo and Salvage Pathway), fatty acid metabolism (e.g., Linolenic acid metabolism), and other lipid metabolism pathways related to acyl chain remodeling (Figure 4E, **Table S7**).

A sex-specific epigenomic association with urate change was also observed three months post-BCG vaccination (long-term change). Moreover, it was notable that there was a high consistency in the effect direction, with males showing 95.5% and females showing 100% consistency between EWAS of short-term urate change and long-term urate change. We identified 33 CpG sites associated with long-term urate change after BCG in females and 112 CpG sites in males (**Figure S4B**), and observed the distinct pattern of effect size between males and females (**Figure S4C**). In males, cg13148076 showed the strongest association with long-term urate change. This probe mapped to *PATZ1*, which plays an important role in regulating T cell development (39). In females, the probe with the greatest association, cg09755770, mapped to gene *RCSD1*, which is involved in the cellular hyperosmotic response (40). We validated these identified long-term urate change-associated CpG sites using an independent cohort (300BCG booster cohort, n=13 females), Thirteen out of 33 CpG sites exhibited the same direction as the discovery cohort and one site (cg04372321, mapped to *DGKQ*) reached to FDR significance (FDR = 0.01) (Figure 4F). *DGKQ* mediates the regeneration of phosphatidylinositol from diacylglycerol in the PI-cycle, further suggesting that the urate change-associated CpG sites were linked to human lipid metabolism.

**Figure 5.**
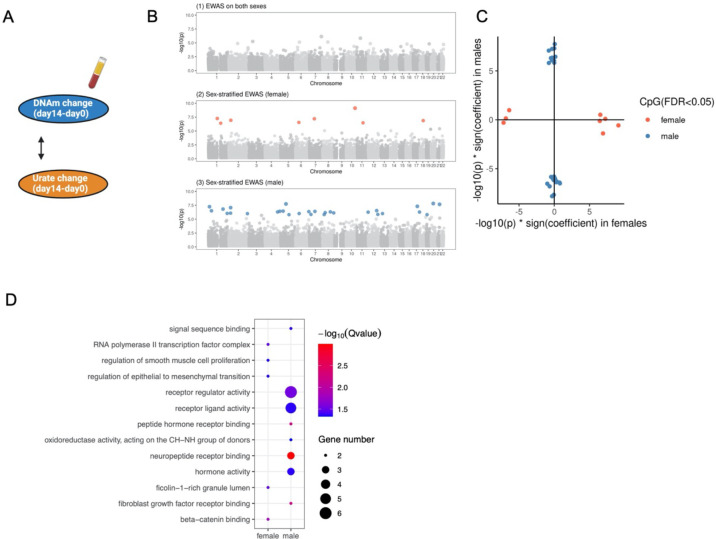
ΔUrate was associated with ΔDNAm upon BCG vaccination in a sex-specific manner. (A) Schematic representation of the association analyses. (B) The Manhattan plot shows the results from the epigenome-wide association between ΔUrate_short_ and ΔDNAm_short_ in all participants (1), females (2), and males (3) respectively. The CpG sites were ordered by their chromosomal position on the x-axis with their −log10 (P value) of the association on the y-axis. Colorful dot: significant CpG sites, grey dot: non-significant CpG sites. (C) The scatter plot showing the distribution of −log10 (P value) * sign(effect estimate) of the significant sites from the epigenome-wide association between ΔUrate_short_ and ΔDNAm_short_ in males and females. The color represented the significant sites from different sex groups. (D) Dot plot describing the KEGG enrichment categories of genes annotated to the significant CpG sites identified from the epigenome-wide association between ΔUrate_short_ and ΔDNAm_short_. The color and size of the dot indicated the significance and the number of annotated genes in each category.

We next investigated the association between urate change (ΔUrate) and DNAm change (ΔDNAm) following BCG vaccination. We first examined the association between short-term urate change ΔUrate_short_ and short-term DNA methylation change ΔDNAm_short_ (Figure 5A). No epigenome-wide association was identified from the EWAS conducted on all participants. However, sex-stratified analyses revealed 29 CpG sites associated with ΔUrate_short_ in males and 8 in females (FDR < 0.05) (Figure 5B). Notably, the strongest association in males was observed for cg00471000 (P value = 1.52e-08), mapping to *BPIFB6* on chromosome 20. In females, the strongest association was seen for cg04660100 (P value = 7.80e-10), mapping to *HABP2* on chromosome 10. Figure 5C depicts the distinctive ΔDNAm_short_-ΔUrate_short_ association profiles between males and females. Functional enrichment analysis of the significant CpG sites in males revealed enrichment for pathways related to receptor regulator activity, receptor-ligand activity, and hormone activity (Figure 5D). We then assessed the association between long-term urate change (ΔUrate_long_) and long-term DNA methylation change (ΔDNAm_long_). Two CpG sites, cg09451092 mapped to *CCDC19* and cg04372321 mapped to *DGKQ*, were identified from the EWAS on all individuals. Similar to the short-term analysis, sex-stratified analysis revealed 52 CpG sites associated with ΔUrate_long_ in males and 6 in females (FDR < 0.05). Males and females exhibited distinct patterns of association (**Figure S5A-B**). Functional enrichment analysis of the significant CpG sites from females indicated a link to the small molecule biosynthetic process (**Figure S5C**).

### Hormones may explain the sex-differential effect, but not CpG sites on chromosome X

To investigate the factors contributing to the observed sex-related differences in urate change after BCG vaccination, we first examined baseline characteristics such as age and body mass index (BMI). However, these factors did not differ significantly between males and females (**Table S1**). Next, we hypothesized that the sex-differential effect may be explained by CpG sites located on sex chromosomes, which are not modeled in our EWAS on autosomes. Therefore, we re-normalized the methylation data by adding data from the X chromosome. Subsequently, we performed association analyses between urate change and CpG sites located on the X chromosome. Following the association analysis, we identified seven CpG sites associated with urate change in females and three in males on the X chromosome, which achieved an X-chromosomal wide FDR significance (**Table S8**). Interestingly, the short-term urate change associated CpG sites on chromosome X in females were annotated to genes related to central nervous system development, axonogenesis and synaptogenesis, and interferon production, such as *ARX*, *SYN1*, *CLCN4*, *MID1*, and *OTUD5*. In males, the most significant long-term urate change associated CpG site was cg11590435, annotated to the *CLCN5* gene, which is involved in the activation of cAMP-dependent PKA and ion channel transport (41). However, when comparing the ratio between X chromosome and autosomes, we did not find significant enrichment on the X chromosome compared to autosomes (P = 0.6 in females, P = 1 in males, Fisher's exact test).

**Figure 6.**
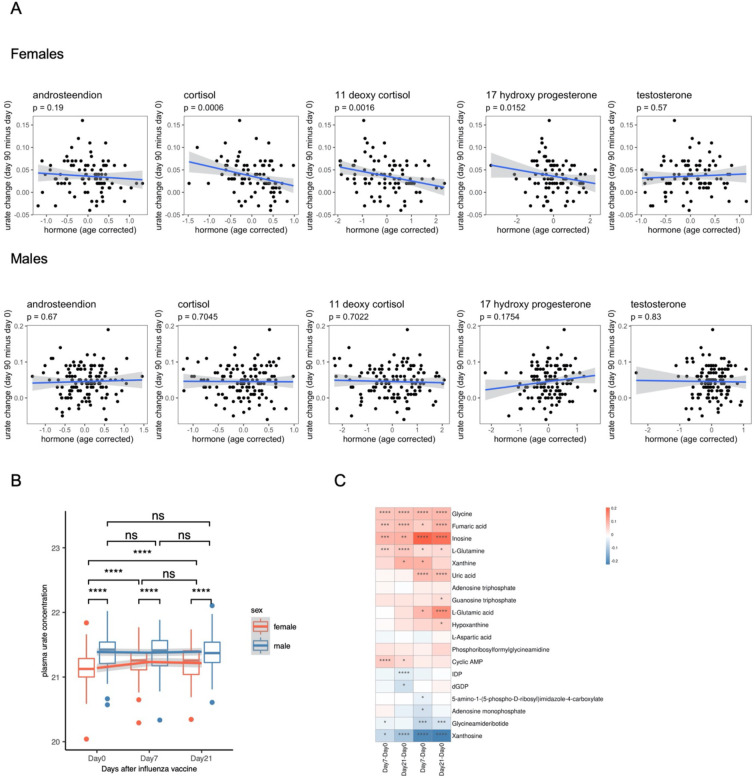
Hormones may modulate sex-differential effect on long-term urate change after BCG and urate increase upon vaccination is not unique to BCG and purine metabolism shift may contribute to. (A) Spearman’s correlation between ΔUrate_long_ and baseline hormone level (after age correction). (B) Boxplot showing the plasma urate concentration at different time points, color indicating different sex groups. The paired samples Wilcoxon test was used to compare the urate level among different time points. Wilcoxon rank sum test was used to compare the difference between males and females. ns: not significant, *: P < 0.05, **: P < 0.01, ***: P < 0.005, ****: P < 0.001. (C) The mean difference of metabolites level between each two time points. These metabolites are all that we can find from the purine metabolism pathway. Stars represented the significance of the difference using paired samples Wilcoxon test. *: P < 0.05, **: P < 0.01, ***: P < 0.005, ****: P < 0.001.

We next investigated whether circulating hormone concentrations might contribute to the sex-specific effects. We analyzed the correlation between baseline hormone concentrations (androstenedione, cortisol, 11-deoxy cortisol, 17-hydroxy progesterone, and testosterone) and baseline urate concentrations or urate change post-vaccination in both males and females. As expected, most of these hormones, except cortisol, displayed sex-based differences in baseline concentrations (**Figure S6A**). Notably, none of these hormones correlated with baseline urate concentrations in either males or females (**Figure S6B**). Intriguingly, baseline plasma cortisol, 11-deoxy cortisol, and 17-hydroxy progesterone showed a negative association with long-term urate change (ΔUrate_long_) after BCG vaccination only in females (cortisol: P value = 0.0006, 11-deoxy cortisol: P value = 0.0016, 17-hydroxy progesterone: P value = 0.0152, Spearman’s correlation), but not in males (Figure 6A). Furthermore, this association was not seen with short-term urate change (ΔUrate_short_) after BCG vaccination (**Figure S6C**).

### Urate increase upon vaccination is not specific to BCG

To determine whether urate increase is specific to BCG vaccination, we analyzed data from an independent cohort of 165 elderly individuals (mean age: 72 years ± 4 SD), who received an adjuvanted trivalent inactivated influenza vaccine (TIV). Plasma metabolome was profiled before vaccination (baseline, day 0), 7 days, and 21 days post-vaccination. Notably, the influenza vaccine significantly increased circulating urate concentration in females, but not males (Figure 6B), suggesting a sex-specific response and a potential mechanism beyond BCG-specific effects. Further investigation of the TIV cohort revealed significant changes in the majority of metabolites within the purine metabolism pathway after vaccination (Figure 6C). These findings suggest that a shift in purine metabolism may contribute to the increased urate levels following vaccination.

## Discussion

In this study, we investigated the sex-specific relationships between DNA methylation and circulating urate concentrations and the effect of BCG vaccination on circulating urate concentrations. We observed significant sex differences in the epigenetic regulation of urate concentrations. Specifically, in males, urate-associated CpG sites were predominantly enriched in genes linked to neuroprotection. Furthermore, we observed an increase in serum urate concentrations post-BCG vaccination in both sexes. Urate change-associated CpG sites in females were linked to lipid and glucose metabolism.

Sex differences in circulating urate concentrations and their association with genetic and cardiometabolic traits are well known. The important role of epigenetic regulation on circulating urate concentration is supported by numerous EWAS findings, such as the recent study from Tin *et al.* (12). However, until now, results from sex-stratified EWAS of urate have not been reported, but are clearly present in our study. For example, one reported CpG site (cg11266682), which is located on gene *SLC2A9* and had a causal effect on urate, presented a significant association with urate only in females (P = 0.0058) but not males (P = 0.62) in our discovery cohort. This finding is consistent with the GWAS results that the genetic variants within this locus were associated with lower serum urate concentrations among women only (42). The urate-associated CpG sites from Tin *et al.* (12) were linked to learning and neurological function, whereas in this study we observed that this pathway was specifically observed in males. The hypothesis that elevated urate is associated with neurodegenerative disease only in males has been proposed in the literature, but evidence remains mixed, with others suggesting that urate showed neuroprotective effects in both sexes (43,44). Our study provides evidence of a common DNA methylation signature of serum urate and neurological function in whole blood specifically in males, though the current results present in this study cannot support the causal effects of serum urate on neuroprotection.

Our study further unveiled the sex-specific epigenetic association between BCG-induced urate change and baseline methylation. The SLC-mediated transmembrane transportation pathway was negatively associated with urate increase exclusively in females. Among the CpG annotated genes enriched in this pathway, *SLC24A3* is a sodium/calcium exchanger. *SLC45A1* and *SLC4A4* play a role in glucose uptake and glucose homeostasis. Additionally, genes related to insulin signaling like *PRKAG2; RHEB; YWHAQ* were positively associated with an increase in urate circulating concentration. These findings suggest the epigenetic co-regulation of urate increases after BCG and glucose metabolism is more prominent in females. The association between higher urate concentrations and the incidence of glucose tolerance, type 2 diabetes development, hypertension development, and lipid accumulation patterns differ between the sexes (45,46). Our findings provide insight that epigenetic co-regulation might be a general mechanism underlying the observed pleiotropy between urate and cardiometabolic traits.

Several pieces of evidence suggest that sex hormones may play a role in sex-dimorphisms of urate. Female hormones are thought to play a role in urate elimination and regulating renal urate transporters (47). In this study, we showed that steroid hormones, including cortisol, 11-deoxy cortisol, and 17-hydroxy progesterone, were associated with the changes in urate concentrations after BCG vaccination (day 90 vs. day 0) in females exclusively. One previous study presented that steroids were necessary to maintain a normal glomerular filtration rate (GFR) and renal plasma flow (RPF) (48). Our results suggest that hormones might engage in controlling and regulating long-term urate increase in females.

The purine metabolite pathway is a potential contributor to the increase in urate concentrations upon BCG vaccination. Previous studies showed that the co-accumulated metabolites module, associated with BCG-induced trained immunity responses, is enriched in the purine metabolism (20). The interplay between epigenetics and metabolites may play an important role in the induction, regulation, and maintenance of trained immunity (49). Furthermore, increased urate leads to immune reprogramming and inflammasome activation, which contribute to the development of inflammation-mediated diseases such as atherosclerosis, reactive arthritis, as well as other autoimmune and autoinflammatory disorders (15,50,51). Our investigation into the effects of influenza vaccination on circulating urate concentration revealed that this urate-elevating phenomenon is not specific to BCG, but rather a more universal feature associated with vaccination and immune stimulation. This is further supported by recent studies reporting that some routinely administered vaccines, including the COVID-19 vaccine and recombinant zoster vaccine, were associated with increased gout flares (13,14,52), thereby pinpointing the possibility of urate concentration increases after the administration of these vaccines.

This study has some limitations. First, the sample size was relatively small, and future studies should involve larger cohorts for validation. Second, although we studied over 800,000 CpG sites of the human methylome, this still only captures about 3% of all human CpG sites (53). Third, our research mainly focused on a few steroid hormones. Further research should explore the role of estrogens and serum parathyroid hormone (PTH) in urate regulation after vaccination. Finally, the epigenome-wide association between whole blood DNA methylation and serum urate concentrations may not represent urate homeostasis in other important organs such as the kidney or liver. Multi-tissue studies might help us to capture a more comprehensive picture of the urate epigenetic regulation.

In conclusion, despite potential limitations, this study reveals sex-specific epigenetic mechanisms underlying urate regulation. These findings improve our understanding of urate biology and may inform the development of sex-specific therapies targeting urate for improved health outcomes.

## Methods

### Cohort information

The 300BCG cohort is a healthy western descent population-based cohort from the Human Functional Genomics Project. Volunteers were recruited between April 2018 and June 2018 in the Radboud University Medical Center as described previously (20,21). All volunteers received a standard dose of 0.1 mL BCG (Intervax, Canada, strain BCG-Bulgaria) intradermally in the left upper arm by a medical doctor. Whole blood was drawn before BCG vaccination (baseline, day 0), 2 weeks (day 14), and 3 months (day 90) after vaccination. Blood was collected in the morning. Exclusion criteria include: acute or chronic illness at the time of sampling; a medical history of immunodeficiency; any febrile illness within 4 weeks before participation; previous BCG vaccination or having lived in tuberculosis endemic countries; history of tuberculosis; any vaccination 3 months before participation; use of systemic medication other than oral contraceptives or acetaminophen; use of antibiotics 3 months before inclusion. This study was approved by the Ethical Committee of Radboud University Medical Center (NL58553.091.16). All participants gave written informed consent.

This study had three replication cohorts, including 500FG, NAS, and BCG booster. 500FG cohort is a part of the Human Functional Genomics Project. Within 500FG, 260 participants (150 females and 110 males) who had both baseline DNA methylation value and circulating urate concentration available were included in this study, with a mean age of 27 years (± 12 standard deviation). NAS (Normative Aging Study, dbGaP Study Accession: phs000853.v1.p1) consists of 774 males with a mean age of 72.65 years (± 6.82 standard deviation). The summary statistic which recorded the EWAS of urate was obtained for the replication analysis. The detailed information of the BCG booster cohort was described previously (27). Within this cohort, 13 females who received single-dose BCG at day0 were included in this study. DNA methylation and serum urate concentrations were measured before and 90 days post BCG vaccination.

The TIV cohort is described in detail previously (54). The main study was conducted between September 2015 to May 2016. 200 individuals (age: 65–80 years) were randomly selected from the residents' registration office in Hannover and received an adjuvanted trivalent inactivated influenza vaccine (TIV).

### DNA methylation quantification and quality control

DNA purification from whole blood was done by QIAamp DNA blood kits (Qiagen Benelux BV, Venlo, the Netherlands). The DNA concentration was measured using a NanoDrop spectrophotometer at 260 nm. High-quality DNA was used for genome-wide DNA methylation profile by either Infinium© MethylationEPIC array (BCG and BCG booster) (~850,000 CpG sites) or MethylationEPIC v2 array (500FG) (~937,055 CpG sites). The DNA methylation values were gained from the raw IDAT files using the minfi package in R (v.4.2.0) (55). We excluded poor quality and sex-mismatched samples. For the probes on the autosomes, stratified quantile normalization (56) was performed after filtering out bad quality probes with a detection P value > 0.01, cross-reactive probes, polymorphic probes (57), and probes on the sex chromosomes. Problematic probes due to mapping inaccuracies and flagged probes in the EPIC v2 array provided by Illumina were also removed in the 500FG cohort. For the probes on the X chromosome, functional normalization was applied (58). After all the quality control steps above, in the BCG cohort, 858 samples (286 individuals for three-time points; females = 160, males = 126), 751,564 probes on the autosome, and 16,724 probes on chromosome X remained for further analyses. In the BCG booster cohort, 26 samples (13 females from two time points) and 794545 probes on autosome remained for further analysis. In the 500FG cohort, we got 296 samples and 854,447 probes on autosomes for further analysis.

### Circulating urate concentration measurement

BCG and BCG booster cohort: Serum was separated from whole blood by centrifuging at 1500 g, 30 minutes. The serum urate concentration was measured by Radboud Laboratory Diagnostic (RLD), with a Roche C8000 system using the module C702. The serum urate value from 935 samples (the number of samples from each time point: baseline: 321; day 14: 314; day 90: 300) was successfully obtained for further analysis.

500FG and TIV cohort: Circulating urate concentrations were obtained from an untargeted metabolism dataset which was described previously (59,60). To confirm if we could use the urate concentration from the untargeted metabolism dataset in the 500FG and TIV cohorts, we did the following analysis: in our 300BCG cohort, we have urate concentrations at baseline from both Radboud Laboratory Diagnostics (RLD) and untargeted metabolism. The values from these two methods were highly correlated (**Figure S7**).

### Epigenome-wide association analyses (EWAS)

Before performing EWAS, we excluded the methylation outliers (value < 25 quantile – 3*IQR or value > 75 quantile + 3*IQR). We performed the EWAS as the previous study did (61): (1) EWAS of baseline urate: To explore how serum urate concentration is associated with methylation, baseline urate concentration was analyzed as the independent variable with M value as the dependent variable in a robust linear regression model adjusting for age, sex, cell type proportions, and batch effect. (M value ~ urate + age + sex + batch + cell type); (2) Sex interaction EWAS: To investigate the sex effect on the association between urate and methylation, we added sex*urate as an interactive factor in our robust linear regression model (M value ~ urate + age + sex + batch + cell type + sex*urate). The P value representing whether 'sex * urate' from the linear model had a significant impact on methylation was recorded; (3) EWAS of urate change after BCG vaccination: To assess the association between baseline methylation and urate change after vaccination, a robust linear model was performed for each CpG site, with rank normalized urate change as the dependent variable, baseline methylation value as the independent variable, age, sex, batch, and cell proportion as the covariates (urate change ~ M value (Day 0) + age + sex + batch + cell type); (4) Sex stratified EWAS: We separated all participants into females and males. The baseline urate-EWAS and urate change-EWAS were conducted in each sex group; (5) Association between urate change and DNAm change: DNA methylation change was the difference in methylation residuals which was calculated by regressing out cell proportion and batch at each time point detailed in our previous publication (62), followed by fitting into the model as: urate change ~ DNAm change + age + sex.

### Downstream analysis

IlluminaHumanMethylationEPICanno.ilm10b4.hg19 R package and Genomic Regions Enrichment of Annotations Tool (63) were used to annotate the identified CpG with their proximal genes. Enrichment analyses were performed using the online tool ConsensusPathDB (CPDB, http://cpdb.molgen.mpg.de) (64). Protein-protein interaction networks functional enrichment analysis was done using STRING (https://string-db.org).

### The association analysis between hormone concentrations and urate change after BCG vaccination

Baseline circulating testosterone, 17-hydroxy progesterone, androstenedione, cortisol, 11-deoxy cortisol were measured by liquid chromatography-tandem mass spectrometry as described previously (65,66). The Spearman method was used to estimate the correlation between hormones and urate change.

### Metabolites from the metabolism pathway in TIV cohort

Metabolome profiling was done using flow-injection mass spectrometry as mentioned previously (60).

## Data Availability

Methylation data and summary statistical results can be accessed via EGAS00001007498 and EWAS Catalog.

## Highlights

- DNA methylation was associated with serum urate in a sex-specific manner.
- Circulating urate concentrations significantly increased after BCG vaccination and elevated urate concentrations persisted for at least three months.
- Baseline DNA methylation was associated with changes in urate concentration after BCG vaccination in a sex-specific manner.
- Urate-associated CpG sites in males were enriched in pathways including neuro-protection.
- CpG sites associated with BCG-induced urate change in females were enriched in pathways related to lipid and glucose metabolism.
